# ABO-incompatible kidney transplantation: impact of apheresis on graft and patient survival in recipients with low isoagglutinin titer

**DOI:** 10.3389/ti.2026.16059

**Published:** 2026-05-26

**Authors:** Marion Mangin, Juliette Lucas, Benjamin Taton, Dany Anglicheau, Gilles Blancho, Dominique Bertrand, Juliette Leon, Marie-Joëlle Apithy, Ilies Benotmane, Lionel Couzi, Sophie Caillard

**Affiliations:** 1 Department of Nephrology Dialysis and Transplantation, Strasbourg University Hospital, Strasbourg, France; 2 Department of Nephrology, Transplantation, Dialysis and Apheresis, Bordeaux University Hospital, Bordeaux, France; 3 INSERM U1219 Bordeaux Population Health, Equipe LEHA, Bordeaux University, Bordeaux, France; 4 Department of Kidney and Metabolic Diseases, Transplantation and Clinical Immunology, Necker Hospital, Assistance Publique - Hôpitaux de Paris, Université Paris Cité, Paris, France; 5 CHU Nantes, Nantes Université, Service de Néphrologie - Immunologie Clinique, ITUN, INSERM, Center for Research in Transplantation and Translational Immunology, UMR 1064, Nantes, France; 6 Nephrology and Transplantation Department, Rouen University Hospital, Rouen, France; 7 Etablissement Francais du Sang Grand Est, Strasbourg, France; 8 Molecular ImmunoRhumatology, INSERM UMR_S1109, Strasbourg University, Strasbourg, France; 9 CNRS-UMR 5164 ImmunoConcEpT, Bordeaux University, Bordeaux, France

**Keywords:** ABO incompatible, antibody mediated rejection, apheresis, delayed graft function, infections

## Abstract

ABO-incompatible (ABOi) kidney transplantation carries a high risk of acute antibody-mediated rejection due to the presence of isoagglutinins. To mitigate this risk, current protocols recommend performing apheresis before transplantation. Our objective was to evaluate outcomes in ABOi recipients with low isoagglutinin titers, comparing those who did and did not undergo pre-transplant apheresis. We conducted a multicenter study including recipients of ABOi kidney transplants between 2012 and 2022. A total of 78 patients with baseline isoagglutinin titers ≤1:8 were included; 41 received pre-transplant apheresis, 37 did not. Patients who did not undergo apheresis had more rejection episodes (p = 0.01), and a trend toward higher rates of delayed graft function and antibody mediated rejection, which adversely impacted patient and graft survival. At 3 years, event-free survival (death or graft loss) was 90% in the apheresis versus 79% in the no-apheresis group (p = 0.02). In multivariable analysis, factors associated with improved event-free survival included pre-transplant apheresis (HR = 0.31, p = 0.049) while ABMR within the first month was associated with poorer outcome (HR = 5.18, p = 0.0007). No differences emerged regarding the occurrence of overall infections. These findings suggest that apheresis should be systematically performed prior to ABOi transplantation, regardless of baseline isoagglutinin titer.

## Graphical Abstract

**Figure:**
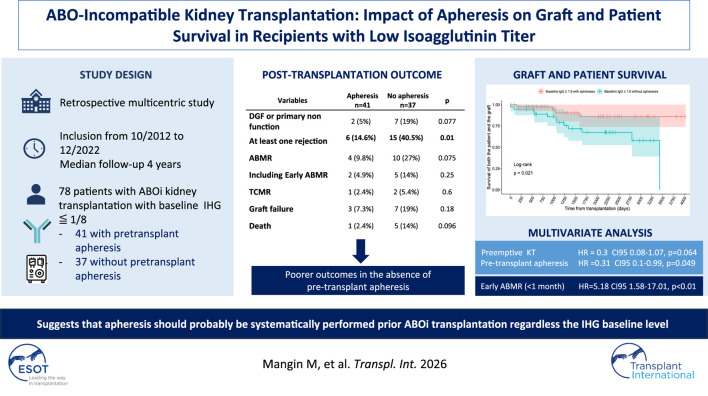

## Introduction

ABO-incompatible (ABOi) kidney transplantation has expanded the living donor pool by overcoming the traditional limitations imposed by ABO blood group compatibility. This strategy has become increasingly relevant in the context of organ shortage and prolonged waiting times, particularly for recipients with less common blood types. In addition to increasing access to transplantation, ABOi kidney transplantation retains the inherent advantages of living donation: elective surgery scheduling, better immunological matching through donor selection, and shorter cold ischemia times. Thanks to advances in immunosuppressive strategies, ABOi transplantation is now routinely performed in many centers. Protocols typically include pre-transplant rituximab administration, and extracorporeal antibody removal—via plasma exchange (PE) or immunoadsorption (IA)—in patients with elevated isoagglutinin titers. Several studies have documented favorable outcomes following ABO-incompatible kidney transplantation, supporting the efficacy of contemporary desensitization strategies [1, 2]. Nonetheless, these findings remain debated, as two meta-analyses reported inferior graft survival in ABOi recipients compared with ABO-compatible controls [3, 4]. ABOi transplantation continues to represent a procedure with substantial immunologic risk, primarily due to the potential for early antibody-mediated rejection (ABMR) driven by anti-A and/or anti-B antibodies, with a relative risk approaching four [4], and occasionally manifesting as severe thrombotic microangiopathy [5]. The relationship between pre-transplant isoagglutinin titers and rejection risk remains controversial: while several investigations have demonstrated a positive correlation between higher titers and increased incidence or severity of rejection [5], others have found no such association [6, 7].

Although a target titer of ≤1:8 on the day of transplantation is frequently used, this threshold varies across institutions. Moreover, in patients with low baseline isoagglutinin levels, the role of apheresis remains controversial. Indeed, despite their potential immunological benefits, apheresis procedures are associated with significant logistical, financial, and clinical burdens. These include the need for central venous access, increased bleeding risk due to coagulation factor depletion, and heightened infection risk. Some studies have also reported higher 1-year mortality and infection rates in ABOi recipients, likely linked to intensified immunosuppressive protocols [1, 4]. Over the past decade, some centers have adopted protocols that omit apheresis when pre-transplant isoagglutinin titers are already below the target threshold, typically ≤1:8. In this context, Masterson [8] reported the outcomes of 20 ABO-incompatible kidney transplants performed at the Royal Melbourne Hospital using a standard immunosuppressive regimen with basiliximab induction but without rituximab nor pre-transplant apheresis, in patients with baseline anti-A and/or anti-B IgG titers ≤1:16 confirmed by three independent techniques. Graft survival at 3 years was 100%, with no significant impairment of graft function. The incidence of ABMR was 5%, comparable to rates reported in studies where isoagglutinin depletion protocols were used.

The aim of our study was to evaluate patient outcome as well as graft and patient survival following ABOi kidney transplantation in a French cohort, in patients with initial low isoagglutinin titers (≤1:8) according to whether or not pre-transplant apheresis was performed. We also assessed the incidence of immunological and infectious complications, to determine whether apheresis conferred any benefit in this low-risk population.

## Patients and methods

For this purpose, we conducted a multicenter retrospective study involving recipients of ABO-incompatible (ABOi) living donor kidney transplants. Participating centers included Necker Hospital (Paris), Strasbourg University hospital, Rouen University hospital, Nantes University hospital, and Bordeaux University hospital. Ethical approval was granted from Bordeaux institutional review board (CER-BDX 2025-338).

### Study population

Eligible patients were all adults who underwent ABO incompatible living donor kidney transplantation with a baseline anti-A and/or anti-B IgG isoagglutinin titer ≤1:8. Transplants were performed between October 2012 and December 2022. Follow-up was completed until October 31, 2023.

### Study design and objectives

Our objectives were to compare: i) graft and patient survival -defined as survival free from graft loss or death-according to the use or absence of pre-transplant apheresis in patients with low baseline IgG isoagglutinin titers (≤1:8); ii) the incidence of immunological complications - ABMR, T cell-mediated rejection [TCMR], borderline rejection- and iii) the occurrence of infectious complications between groups. We also aimed to identify risk factors associated with graft and patient survival.

### Data collection

Clinical data were retrospectively extracted from medical records—both paper and electronic—using local hospital systems: Sined and DxCare (Strasbourg), Orbis (Necker), Sined (Rouen), Millenium and DIVAT database (Nantes), DxCare and R@N (Bordeaux). Isoagglutinin titers were provided by the French Blood Establishment (EFS). We recorded rituximab administration (date and dose), induction therapy (anti-thymocyte globulin (ATG) or basiliximab), and maintenance immunosuppression at hospital discharge (tacrolimus, cyclosporine, mycophenolate mofetil [MMF], corticosteroids). The number and type of apheresis sessions (PE, IA, or double filtration plasmapheresis [DFPP]) performed pre- and post-transplant were documented. The use of intravenous immunoglobulin (IVIg) in the post-transplant period was also recorded. Presence of preformed or *de novo* donor specific antibodies (DSA) was recorded considering a MFI threshold of 1000 (Luminex assay, Lab. One lambda, in all centers).

### Isoagglutinin titers

Anti-A and/or anti-B IgG and IgM titers were measured at baseline (prior to desensitization), on the day before or the day of transplantation (pre-transplant titer), and post-transplant at week 1, month 1, month 3, and month 12, as well as at the time of any suspected rejection. For final analysis, only IgG titers were considered. Techniques used for isoagglutinin measurement varied by center: tube hemagglutination at Necker, gel hemagglutination at Rouen, Strasbourg, Bordeaux and Nantes.

### Pre-transplant desensitization

Rituximab (a single dose of 375 mg/m^2^) was administered before transplantation (day −30) in all but four patients, according to local protocols. Apheresis sessions were performed according to local procedures. It was systematically applied in Strasbourg (9 patients, treated with 3 sessions) and in the majority of patients in Necker (24/28 patients treated with 1–13 sessions; mean 3.3 sessions). In contrast, apheresis was generally not used in Rouen (6/9 patients without apheresis), Bordeaux (9/11 patients without apheresis), or Nantes (15/18 patients without apheresis).

The apheresis techniques used were as follows: Glycorex immunoadsorption (n = 5) and plasma exchange (n = 4) in Strasbourg; plasma exchange in Necker (n = 23), except for one patient with preformed DSA treated with immunoadsorption (IA); plasma exchange in Bordeaux (n = 2) and Nantes (n = 3); and immunoadsorption (IA, Globaffin®) in Rouen (n = 3). Additional details regarding local procedures are provided in Supplementary Table S1.

### Post-transplant follow-up

Delayed graft function (DGF) was defined as the need for at least one dialysis session during the first post-transplant week. Follow-up variables included serum creatinine and glomerular filtration rate according to CKD EPI formula at 3, 12, and 36 months, graft function status at end of follow-up, date of return to dialysis (if applicable), patient death (date and cause: cardiovascular, oncologic, or infectious), and biopsy-proven rejection episodes (classified according to Banff criteria). The presence of *de novo* DSA, including MFI and class, and isoagglutinin titer at the time of rejection, were also recorded. ABMR was defined as follows: immediate cortical necrosis consistent with hyperacute rejection occurring within hours after transplantation, early thrombotic microangiopathy, or histological findings consistent with ABMR according to the Banff classification on graft biopsy performed later post-transplant. Early ABMR was defined as ABMR rejection occurring during the first post-transplant month.

For infectious complications, the type and date of each event were collected. In cases of recurrent infections of the same type, only the first occurrence was dated. Events occurring after graft loss were not included in the analysis.

### Statistical analysis

All statistical analyses were performed using R software (version 4.3.2) and appropriate packages. Quantitative variables with a symmetrical distribution were summarized as mean ± standard deviation (SD), while non-normally distributed quantitative variables and ordinal data were presented as median [first quartile; third quartile]. Categorical variables were expressed as counts (percentage). A p-value <0.05 was considered statistically significant. Group comparisons were conducted using Fisher’s exact test for categorical variables and the Kruskal–Wallis test for continuous variables. Survival analyses were performed using a composite endpoint defined as “graft loss or patient death.” Event-free survival was estimated using Kaplan–Meier curves, and differences between groups were assessed with the log-rank test. For the analysis of rejection events, cumulative incidence functions were estimated using the Aalen-Johansen estimator, accounting for graft loss or death as competing events. Between-group comparisons were performed using Gray’s test. Risk factors associated with ABMR, biopsy-proved rejection episodes (BPAR: ABMR + TCMR), and a composite endpoint of graft loss or death were investigated using Cox proportional hazards models. Cox regression results are reported as hazard ratios (HR) with 95% confidence intervals (95% CI). Comparisons of HRs to the null value (HR = 1) were assessed using Wald tests. Explanatory variables were first assessed in univariate analyses including all patients with available data. A multivariable Cox model was then constructed, incorporating variables that were statistically associated with the outcome in univariate analysis and those considered clinically relevant (p ≤ 0.2). In addition, we performed a LASSO-Cox regression to assess the association of the covariables with death and graft failure. The regularization coefficient was selected using 5-fold cross-validation as the one that optimized the C-statistics [REF glmnet]. Stability of variable selection was assessed by bootstrapping the whole procedure (1000 samples) then computing the proportion of samples for which each variable was selected by the LASSO-Cox regression.

## Results

### Patient characteristics

A total of 78 patients with baseline anti-A and/or anti-B IgG isoagglutinin titers ≤1:8 were included (see characteristics in Table 1). The cohort was predominantly male (69%). Preemptive transplantation was performed in 45% of cases. Prior to transplantation, 58% of patients (n = 45) were sensitized and 6 (7.7%) had also preformed DSA with a median sum of MFI of 1400 (range 1000–6500). Blood group O was the most common among recipients (46%). Donors were predominantly female (71%), with blood group A being the most frequent (63%), followed by group B (26%) and AB (12%). The most common donor-recipient combination was from a group A donor to a group O recipient, accounting for 36% of cases. Most patients (94%) received anti-CD20 monoclonal antibody therapy prior to transplantation.

**TABLE 1:** Baseline characteristics of 78 kidney transplantation recipients with a baseline isoagglutinin titers ≤1:8 and comparison between groups according to pre-transplant apheresis sessions. Mean ± SD, median [IQR], number (%).

Variables	Total cohort n = 78	Patients with apheresis n = 41	Patients without apheresis n = 37	P*
Recipient age (year)	50.37 ± 13.8	50.04 ± 13.81	50.73 ± 13.98	0.83
Recipient sex (M/F)	54 (69%)/24(31%)	29 (71%)/12 (29%)	25 (68%)/12 (32%)	0.81
Nephropathy	​	​	​	0.45
CTIN	30 (38%)	17 (41%)	13 (35%)	​
Glomerulopathy	22 (28%)	10 (24%)	12 (32%)	​
APKD	14 (18%)	8 (20%)	6 (16%)	​
Vascular nephropathy	7 (9%)	2 (5%)	5 (14%)	​
Other	5 (6.4%)	4 (10%)	1 (3%)	​
Preemptive transplantation (yes/no)	35 (45%)/43(55%)	17 (41%)/24 (59%)	18 (49%)/19 (51%)	0.65
Graft rank	​	​	​	0.22
1	65 (83%)	35 (85%)	30 (81%)	​
2 or more	13 (16.6%)	6 (15%)	7 (19%)	​
Preformed DSA (yes/no)	6 (7.7%)/72(92.3)	4 (9.7%)/37 (90.3%)	2 (5.4%)/35 (94.6%)	0.68
Blood type incompatibility	​	​	​	0.76
A > B	21 (27%)	12 (29%)	9 (24%)	​
A > O	28 (36%)	17 (41%)	11 (30%)	​
AB > A	6 (7.7%)	3 (7%)	3 (8%)	​
AB > B	3 (3.8%)	1 (2%)	2 (5%)	​
B > A	12 (15%)	5 (12%)	7 (19%)	​
B > O	8 (10%)	3 (7%)	5 (14%)	​
Rituximab (yes/no)	73 (94%)/5(6.4%)	40 (98%)/1 (2.4%)	33 (89%)/4 (11%)	0.18
Baseline IHG titer	​	​	​	0.18
0 or 1:1	20 (25.6%)	7 (17%)	13 (35.1%)	​
1:2 or 1:4	37 (47.4%)	21 (51.2%)	16 (43.2%)	​
1:8	21 (26.9%)	13 (31.7%)	8 (21.6%)	​
Pre transplant apheresis	​	​	​	​
PE	31 (40%)	31 (40%)	—	​
IA	6 (7.7%)	6 (7.7%)	—	​
IA + PE	3 (3.8%)	3 (3.8%)	—	​
PE + DFPP	1 (1.3%)	1 (1.3%)	—	​
Pre transplant apheresis number	3 [2, 5]	3 [2, 5]	—	​
Pre transplant IA number (n = 9)	3 [2, 5]	3 [2, 5]	—	​
Pre transplant PE number (n = 35)	3 [1, 5]	3 [1, 5]	—	​
IHG titer at time of transplantation	​	​	​	0.19
0 or 1:1	34 (43.5%)	22 (53.6%)	12 (32.4%)	​
1:2 or 1:4	29 (31.2%)	12 (29.3%)	17 (45.9%)	​
1:8	13 (17%)	6 (14.6%)	7 (18.9%)	​
1:16	1 (1.3%)	0 (0%)	1 (3%)	​
Missing data	1 (1.3%)	1 (2.4%)	—	​
Donor sex (M/F)	23 (29%)/55(71%)	12 (29%)/29 (71%)	11(30%)/26 (70%)	1
Donor age (year)	53.95 ± 12.23	54.2 ± 11.51	53.68 ± 13.14	0.85
Donor GFR (mL/min)	94.68 ± 13.31	92.79 ± 13.28	96.76 ± 13.21	0.19
Induction: basiliximab/ATG	34 (44%)/44(56%)	27 (66%)/14 (34%)	17 (46%)/20 (54%)	0.11
CNI: Tacrolimus/Cyclosporine	76 (97%)/2(2.6%)	39 (95%)/2 (5%)	37 (100%)/0 (0%)	0.49
MMF (yes/no)	74 (95%)/4(5.1%)	41 (100%)/0 (0%)	33 (89%)/4 (11%)	0.046
IVIg (yes/no)	26 (33%)/52(67%)	21 (51%)/20 (49%)	5 (14%)/32 (86%)	0.00062

CTIN, chronic tubulo interstitial nephropathy; APKD, autosomal Polycystic Kidney Disease; DSA, donor specific antibodies; PE, plasma exchange; IA: immunoadsorption; DFPP, double filtration plasmapheresis; GFR, glomerular filtration rate; IHG, Isohemagglutinin (IgG only); ATG, Anti-Thymocyte Globulin; CNI, calcineurin inhibitor; MMF, mycophenolate mofetil; IVIg, Intravenous Immunoglobulin.

* comparisons between groups with or without apheresis.

Among the 41 patients (53%) who did undergo apheresis, PE alone was most commonly used (n = 31), followed by IA (n = 6), and a combination of PE and IA (n = 3). One patient received a combination of PE and DFPP. On average, patients who underwent apheresis received 3 sessions (range: 2–5). In contrast, pre-transplant apheresis was not performed in 37 patients (47%).

The main baseline differences between patients who did and did not undergo pre-transplant apheresis are summarized in Table 1. Of note, one of the 37 patients with a baseline IgG ≤1:8 who did not undergo apheresis showed an increase in IgG titer to 1:16 on the day of transplantation. Briefly, patients in the no-apheresis group were less often treated with IVIG and MMF (p < 0.01 and 0.046 respectively).

### Post transplant outcome

In the whole cohort, patient survival after transplantation was 99.5% at 1 year, 97% at 3 years, and 95.5% at 5 years and graft survival was 94.3% at 1 year, 90% at 3 years, and 86% at 5 years.

**FIGURE 1:**
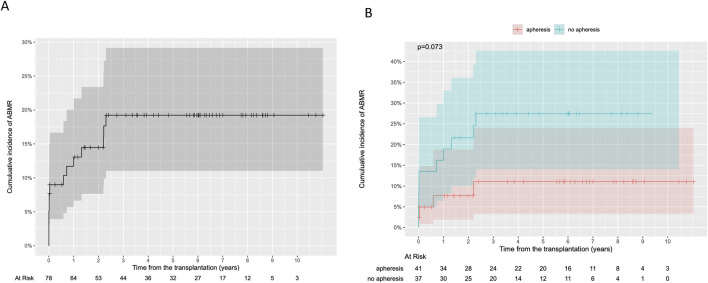
**(A)** Cumulative incidence of ABMR in the whole cohort (n = 71) **(B)** Antibody mediated rejection cumulative incidence according to pre-transplant apheresis status. Patients who did not undergo apheresis had a trend toward more ABMR episodes (p = 0.073, log-rank test). Patients with apheresis, n = 41, red curve and patients without pretransplant apheresis, n = 37, blue curve.

A total of eight patients experienced delayed graft function and one patient displayed primary non-function (Table 2). Only 4 patients showed a IHG rebound (defined as a post-transplant IgG titer ≥1:16) in the first post-transplant month. During follow-up, we recorded 22 rejection episodes which occurred in 21 patients, including 14 ABMR, 3 TCMR, and 5 borderline rejections. Among them, 7 patients developed early acute ABMR, most cases occurring immediately after transplantation. Their characteristics are described in Supplementary Table S2. During follow up, 11 patients developed *de novo* DSA, after a median delay of 15.3 months (range 0.5–83 months). Cumulative incidence of ABMR was 12% at 1 year, and 19% at 3 and 5 years (Figure 1A). Ten patients lost their graft (see Supplementary Table S3) and 6 died. Median follow-up time after transplantation was 4.29 years [2.22, 6.72].

**TABLE 2:** Post transplantation outcome of the whole population, and in the 2 groups of patients, according to pretransplant apheresis sessions. Median [IQR], number (%).

Variables	Total Cohort, n = 78	Patients with apheresis, n = 41	Patients without apheresis, n = 37	P*
DGF or primary non function	​	​	​	0.077
No	69 (88%)	39 (95%)	30 (81%)	​
Yes	9 (12%)$	2 (5%)	7 (19%)$	​
Post transplant apheresis	​	​	​	0.24
none	65 (83.3%)	33 (80.5%)	32 (86%)	​
PE	10 (16.7%)	5 (12.2%)	5 (14%)	​
Missing data	3 (3.9%)	3 (7.3%)	0	​
Number of post-transplant PE sessions	5 [3; 5]	5 [3; 5]	4 [2; 5]	0.67
IHG titer ≥1:16 during the first month post-transplant (%)	4/68 (5.9%)	3/37 (8.1%)	1/31 (3.2%)	0.39
*De novo* donor-specific antibody	11 (14.1%)	6 (14.6%)	5 (13.5%)	0.88
At least one rejection (yes/no)	21(26.9%)/57(73.1%)	6(14.6%)/35 (85.4%)	15(40.5%)/22(59.5)	0.01
ABMR (yes/no)	14 (17%)/65(83%)	4 (9.8%)/37 (90%)	10 (27%)/27 (73%)	0.075
Including early ABMR (Yes/No)	7 (9%)/71 (91%)	2 (4.9%)/39 (95%)	5 (14%)/32 (86%)	0.25
TCMR (yes/no)	3 (3.8%)/75(96%)	1 (2.4%)/40 (98%)	2 (5.4%)/35 (95%)	0.6
Borderline rejection (yes/no)	5 (6.4%)/73(94%)	1 ((2.4%)/40 (97.6%)	4 (10.8%)/33 (89.2%)	0.18
Graft failure (yes/no)	10 (13%)/68(87%)	3 (7.3%)/38 (93%)	7 (19%)/30 (81%)	0.18
Death (yes/no)	6 (7.7%)/72(92%)	1** (2.4%)/40 (98%)	5*** (14%)/32 (86%)	0.096

DGF, delayed graft function; PE, plasma exchange; ABMR, Antibody Mediated Rejection; Early ABMR, ABMR, rejection occurring during the first post-transplant month; TCMR, T-cell Mediated Rejection.

^$^1 patient with primary non function.

* comparisons between groups with or without apheresis.

** from neoplasia.

*** 1 from cardiovascular cause (heart rhythm disorder), 1 from infectious cause (pneumocystis) and 3 due to neoplasia.

Comparison of outcomes according to pre-transplant apheresis status is shown in Table 2. In the group without apheresis, one patient experienced graft loss due to cortical necrosis at the time of transplantation, and four patients developed hyperacute rejection with thrombotic microangiopathy. Consistent with these findings, there was a trend toward a higher incidence of DGF in patients who did not receive apheresis compared to those who did (19% vs. 5%, p = 0.077). In 5 out of 8 patients with DGF (four in the no-apheresis group and one in the apheresis group), DGF was attributed to thrombotic microangiopathy and/or hyperacute rejection. The evolution of isoagglutinin titers during the first post-transplantation year according to the groups is depicted in Supplementary Figure S1 and showed a rebound in 3 patients of the apheresis group and 1 in the no-apheresis group. Post transplant apheresis were performed in 5 patients of each group, according to local center procedures, due to isoagglutinin rebound (n = 3) and/or rejection episodes (n = 7).

**FIGURE 2:**
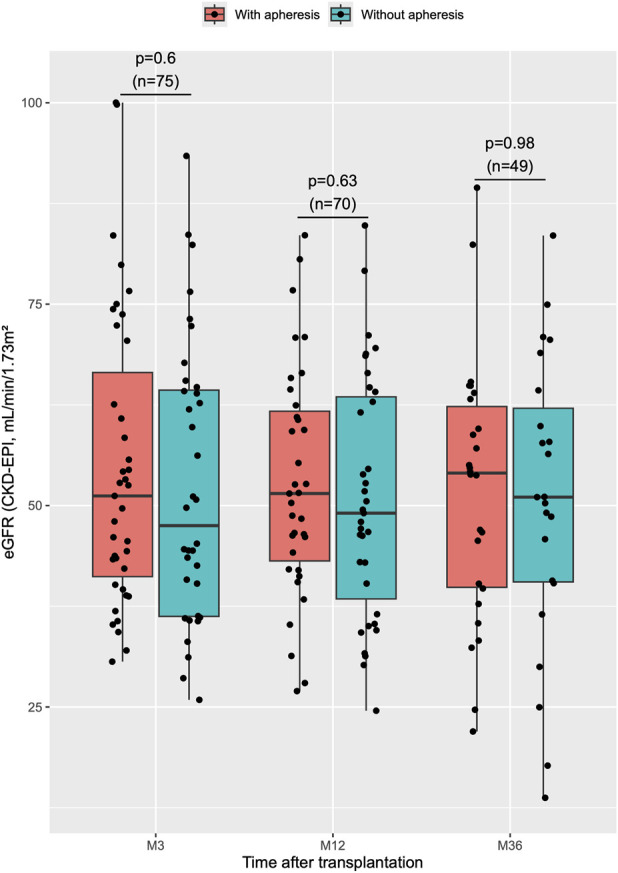
Evolution of eDFG (CKD EPI formula) in the 3 post-transplant years, in patients with a functioning graft, in apheresis group (red plots) compared to the no-apheresis group (blue plots).

Rejections episodes were more frequent in the no-apheresis group (40.5% vs. 14.6%, p = 0.01). Early ABMR episodes were also more frequent in the no-apheresis group (14% vs. 4.9%), although the difference did not reach statistical significance (p = 0.25). Of note, one of the two early ABMR episodes in the apheresis group occurred in a patient with high preformed donor-specific antibodies (DSA) with a cumulated MFI >6000. Kaplan–Meier analysis similarly demonstrated a trend toward a higher incidence of ABMR episodes in patients who did not receive apheresis (p = 0.073, Figure 1B). Evolution of glomerular filtration rate in both group during the study is depicted in Figure 2 and was not different between groups.

### Patient and graft survival

**FIGURE 3:**
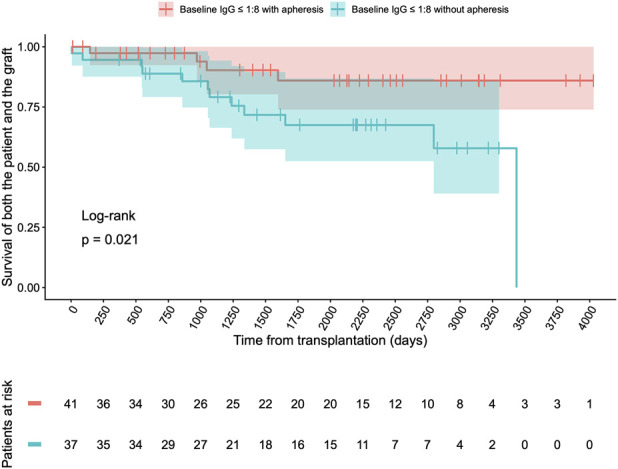
Event-free survival (graft survival and patient survival) according to pre-transplant apheresis status. Patients who did not undergo apheresis had significantly lower event-free survival (p = 0.021, log-rank test). Patients with apheresis, n = 41, red curve and patients without pretransplant apheresis, n = 37, blue curve.

**FIGURE 4:**
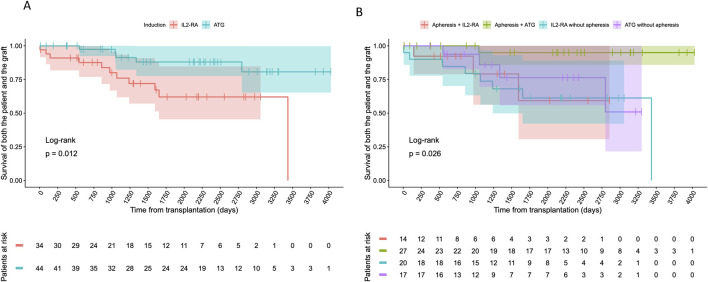
**(A)** Event-free survival stratified by induction therapy. Thymoglobulin was associated with significantly better outcomes compared to Basiliximab; Basiliximab, n = 34, red curve and ATG, n = 44, blue curve. **(B)** Event-free survival by combined stratification of pre-transplant apheresis and induction regimen. The subgroup receiving both apheresis and thymoglobulin induction showed the highest survival rates (p = 0.026, log-rank test): ATG + apheresis, n = 27 green curve; ATG without apheresis, n = 17, purple curve; Basiliximab + apheresis, n = 14, red curve; and Basiliximab without apheresis, n = 20, blue curve.

A significant difference in event-free survival—defined as survival free from graft loss or all-cause mortality—was observed comparing patients who underwent pre-transplant apheresis and those who did not (p = 0.021), with inferior outcomes in the no-apheresis group (Figure 3). At 3 years, event-free survival (death or graft loss) was 90% in the apheresis group and 79% in the no-apheresis group. At 5 years, it was 86% in the apheresis group and 68% in the no-apheresis group. When event-free survival was analyzed according to baseline IHG titer strata, no significant differences were observed between groups (Supplementary Figure S2). Subsequent analysis based on the induction regimen revealed that patients receiving thymoglobulin (n = 44) demonstrated superior event-free survival compared to those treated with Basiliximab (n = 34) (Figure 4A). When stratifying both apheresis status and induction therapy, the subgroup receiving pre-transplant apheresis in combination with thymoglobulin induction exhibited the most favorable outcome (p = 0.026) (Figure 4B).

When death and graft loss were analyzed separately, a higher number of events was observed in the group that did not undergo pre-transplant apheresis, but these differences did not reach statistical significance (p = 0.07 for mortality and p = 0.19 for graft loss, Supplementary Figure S3). Precise causes of graft loss are provided in Supplementary Table S3. When comparing outcomes based on induction therapy, graft loss was significantly less frequent among patients who received thymoglobulin compared to those who received basiliximab (p = 0.012, Supplementary Figure S4), while no significant difference was observed in mortality between the two groups (p = 0.61, data not shown).

### Factors associated with patient and graft survival and rejection episodes (AMR and BPAR)

Factors significantly associated with improved patient and graft survival in univariate analysis were the use of pre-transplant apheresis (HR = 0.28, 95% CI 0.091–0.89, p = 0.03) and thymoglobulin induction therapy (HR = 0.28, 95% CI 0.096–0.81, p = 0.019). Conversely, delayed graft function and early ABMR were linked to an increased risk of graft loss and patient death (HR = 3.3, 95% CI 1.1–10, p = 0.041 and HR = 6.9, 95% CI 2.1–22, p = 0.0012, respectively). The presence of preformed donor-specific antibodies was not statistically associated with outcomes in this cohort. These findings are summarized in Table 3. In multivariable Cox regression analysis, pre-transplant apheresis was independently associated with improved patient and graft survival (HR = 0.31, 95% CI 0.10–0.99, p = 0.049). Conversely, the occurrence of early ABMR (within the first month post-transplant) was significantly associated with an increased risk of patient death and graft loss (HR = 5.18 95% CI 1.58–17.01, p = 0.0007). These results are summarized in Table 4. The variables selected by the LASSO model corroborate those identified in the multivariate Cox model (Supplementary Figure S5).

**TABLE 3:** Factors associated with event free survival (graft survival and patient survival) in 78 kidney transplant recipients with low (≤1:8) IHG in univariate analysis.

Variables	HR	CI95	p
Recipient age	1	[0.97; 1]	0.81
Recipient sex (F vs. M)	0.47	[0.13; 1.6]	0.24
Preemptive transplantation (yes vs. no)	0.35	[0.099; 1.2]	0.10
First graft (yes vs. no)	1.87	[0.42; 8.3]	0.41
A to O transplantation (vs. other incompatibilities)	1.56	[0.58; 4.2]	0.38
Sensitized (yes vs. no)	0.64	[0.23; 1.8]	0.40
Preformed DSA (yes vs. no)	2.49	[0.56; 11.09]	0.23
Rituximab (yes vs. no)	0.36	[0.08; 1.6]	0.18
**Pre transplant apheresis (yes vs. no)**	**0.28**	**[0.091; 0.89]**	**0.03**
Donor age	1	[0.96; 1]	0.93
Donor sex (F vs. M)	3	[0.69; 13]	0.14
Donor eGFR	1.02	[0.99; 1.1]	0.18
IgG at D0			
<1:8	ref.	-	-
≥1:8	0.63	[0.14; 2.8]	0.54
**Induction (ATG vs. basiliximab)**	**0.28**	**[0.096; 0.81]**	**0.019**
**DGF (Yes vs. No)**	**3.3**	**[1.1; 10]**	**0.041**
**Early ABMR (yes vs. no)**	**6.9**	**[2.1; 22]**	**0.0012**

CTIN: chronic tubulo-interstitial nephropathy; APKD, autosomal polycystic Kidney Disease; eGFR, estimated Glomerular Filtration Rate; ATG, anti thymoglobulin; DGF, delayed graft function; PNF, primary non function; ABMR, antibody mediated rejection; DSA, donor specific antibodies; D0, day of kidney transplantation.

**TABLE 4:** Multivariate analysis of factors associated with event free survival (graft survival and patient survival) in 78 kidney transplant recipients with low IHG (IgG≤1:8).

Covariable	HR	CI95	p
Preemptive transplantation	0.30	[0.08; 1.07]	0.064
Pre transplant apheresis	0.31	[0.10; 0.99]	0.049
Early ABMR	5.18	[1.58; 17.01]	0.0007

Early ABMR , antibody mediated rejection in the first post transplant month.

Regarding rejection episodes, we observed a trend toward an association between pre-transplant apheresis and ABMR or BPAR (p = 0.08 and 0.09, respectively), in both univariate and multivariate analyses (Supplementary Tables S4, S5).

### Infections

No differences were observed between groups with or without pre-transplant apheresis considering occurrence of all infections. The probability of viral, bacterial and fungal infections at 1- and 3-year post-transplantation according to apheresis group are described in Table 5. There were more fungal or parasitic infections in the no apheresis group (p = 0.047) −1 microsporidiosis *vs.* 2 pneumocystis pneumonia, 2 candidiases and 3 cryptosporidioses-, and a trend toward a higher incidence of CMV infection among patients who underwent apheresis (p = 0.056; Supplementary Figure S6). Presumed BKV nephropathy incidence was not different between the two groups.

**TABLE 5:** Probability of infections occurrence at 1- and 3-year post-transplantation according to pre transplant apheresis or not.

Infection	Time	Apheresis group (n = 41)	No apheresis group (n = 37)	p
Any infection				0.58
​	1 year	42 [26, 57]	32 [18, 48]	​
​	3 years	59 [40, 74]	53 [35, 68]	​
Any viral infection				0.12
​	1 year	24 [11, 38]	14 [4.8, 27]	​
​	3 years	37 [21, 53]	23 [10, 38]	​
Presumptive BKV nephropathy				0.39
​	1 year	16 [6.3, 29]	11 [3.4, 23]	​
​	3 years	19 [8.2, 33]	11 [3.4, 23]	​
CMV disease				0.17
​	1 year	0	0	​
​	3 years	0	5.7 [0.99, 17]	​
CMV infection				0.056
​	1 year	21 [9.5, 35]	2.7 [0.2, 12]	​
​	3 years	23 [11, 38]	8.2 [2.1, 20]	​
Viral pneumonia				0.086
​	1 year	5.2 [0.9, 15]	2.7 [0.2, 12]	​
​	3 years	12 [3.5, 25]	5.9 [1, 17]	​
Norovirus				0.52
​	1 year	2.6 [0.2, 12]	0	​
​	3 years	5.7 [0.98, 17]	3.2 [0.22, 14]	​
Any bacterial infection				0.83
​	1 year	23 [11, 38]	22 [10, 36]	​
​	3 years	37 [21, 53]	39 [23, 55]	​
Bacterial pneumonia				0.72
​	1 year	2.4 [0.18, 11]	2.7 [0.2, 12]	​
​	3 years	8.6 [2.1, 21]	8.6 [2.1, 21]	​
Pyelonephritis				0.89
​	1 year	18 [7.9, 32]	19 [8.2, 33]	​
​	3 years	18 [7.9, 32]	31 [16, 46]	​
Bacteriemia				0.14
​	1 year	7.8 [2, 19]	2.7 [0.2, 12]	​
​	3 years	15 [5.1, 29]	5.5 [0.96, 16]	​
Any fungal or parasitic infection				0.047
​	1 year	0	5.4 [0.94, 16]	​
​	3 years	0	11 [3.5, 24]	​
Invasive fungal infection (excluding pneumocystosis)				0.15
​	1 year	0	2.7 [0.2, 12]	​
​	3 years	0	2.7 [0.2, 12]	​
Parasitic infection				0.31
​	1 year	0	0	​
​	3 years	3 [0.22, 14]	5.9 [1.17]	​
Pneumocystis infection				0.32
​	1 year	0	2.7 [0.2, 12]	​
​	3 years	0	2.7 [0.2, 12]	​

[Confidence interval 95%].

Between-group comparisons were performed using Gray’s test.

## Discussion

This multicenter French study examined the outcomes of 78 ABO-incompatible kidney transplant recipients with baseline anti-A and/or anti-B IgG titers ≤1:8, over a median follow-up of 4 years. In this cohort, both patient and graft survival rates were excellent, with patient survival at 99.5% at 1 year, 97% at 3 years, and 95.5% at 5 years, and graft survival at 94.3% at 1 year, 90% at 3 years, and 86% at 5 years. However, patients who did not undergo pre-transplant apheresis had poorer outcomes, with lower event-free survival (defined as survival free from graft loss or death), a higher rate of rejection, and a trend toward higher rates of DGF and ABMR.

Overall, patient and graft survival in our cohort appears higher than that reported in studies including ABO-incompatible transplant recipients regardless of baseline isoagglutinin titers. In the study by Montgomery et al. [9], patient survival was 93.7% at 3 years and 88.3% at 5 years, while in the cohort reported by Barnett et al. [10], 1- and 3-year survival rates were 94.5% and 91.9%, respectively. In contrast, our results align with a single-center study from Spain [11], which included 57 patients with low baseline isoagglutinin titers (<1:16), of whom 50% did not undergo pre-transplant apheresis. In that study, patient survival was 100% at both 1 and 5 years, and graft survival was 90% at both time points. However, patients in that cohort were younger and less sensitized than those in our study.

In our cohort, patients who did not undergo pre-transplant apheresis had poorer outcomes compared to those who did, with reduced event-free survival and a marked trend toward higher ABMR incidence. We observed one case of primary non-function due to cortical necrosis in the no-apheresis group, and six additional early ABMR episodes with thrombotic microangiopathy (TMA) within the first month, of which four occurred in the no-apheresis group. It should also be emphasized that the two groups were not fully comparable, as patients in the no-apheresis group generally received less intensive immunosuppression, including lower use of rituximab, MMF, and ATG induction therapy.

Studies investigating ABO-incompatible kidney transplantation without pre-transplant apheresis in patients with low isoagglutinin titers have also reported cases of hyperacute rejection. Shinoda et al. [12] described one episode of hyperacute rejection with TMA and thrombosis among 35 patients without apheresis. In the study by Masterson [8], one hyperacute rejection with TMA was reported among 20 ABOi transplantations performed without apheresis, although the graft was successfully salvaged by post-transplant apheresis. Nevertheless, these rare events did not result in statistically significant differences in graft survival across most studies. For instance, the Japanese study by Kawamura [13] compared ABOi transplants without apheresis to historical ABO-compatible transplants in a pediatric population with baseline isoagglutinin titers ≤1:64. Rituximab was administered at 100 mg twice, and induction therapy was done with basiliximab. No differences in patient survival, graft survival, or rejection rates were observed.

In these studies, basiliximab induction was used, and the potential benefit of depleting induction therapy in reducing the risk of rejection was not evaluated. In our cohort, the subgroup of patients who received both pre-transplant apheresis and ATG induction had the most favorable outcomes and the lowest incidence of graft loss. The benefit of a depletant induction in this context was also underlined in a recent study from Netherlands [14]. Of course, this must be weighed against the theoretical increased risk of infectious and neoplastic complications associated with such intensive immunosuppression.

In our cohort, univariate analysis identified depleting induction therapy and pre-transplant apheresis as factors associated with improved patient and graft survival, while the occurrence of hyperacute or acute ABMR was, as expected, associated with poorer outcomes. The association between DGF and worse prognosis is likely explained by the high frequency of ABMR with thrombotic microangiopathy, which contributed to DGF in most cases. In multivariable analysis, pre-transplant apheresis and early rejection remained independently associated with event-free survival, supporting the potential role of pre-transplant apheresis in reducing the risk of very early immunologic or thrombotic events.

The “protective” role of apheresis in reducing the risk of acute ABMR and improving graft survival in patients with low baseline isoagglutinin titers remains a matter of debate. One hypothesis is that hemagglutination-based assays, commonly used to measure antibody levels, may lack sufficient sensitivity or specificity to accurately reflect the true antibody burden. In the UK, centers showed up to a five-dilution titer difference in National External Quality Assessment Scheme data comparing in-house techniques across laboratories. This finding highlights significant variability, where a single titer result could be interpreted as low in some centers and high in others [15]. Moreover, blood groups A and B are classified into six ABH subtypes. Using an ABH-glycan microarray, studies have reported a poor correlation between haemagglutination IgG titers against A red blood cells and IgG binding to individual subtypes I through VI. Since the subtype II antigen is predominantly expressed on renal vascular endothelium, identifying IgG binding specifically to subtype II may be more clinically relevant [16]. Finally, the respective roles of immune (IgG) and non-immune (IgM) isoagglutinin in the pathogenesis of rejection are not clearly defined [6], and only IgG titers were used in our final analysis. Interestingly, in our cohort, 39 out of 46 patients with available baseline IgM data also had titers ≤1:8, and six of the seven patients with IgM titers >1:8 received pre-transplant apheresis. Notably, the single patient with an IgM titer of 1:32 who did not receive apheresis developed cortical necrosis and primary non-function, likely due to hyperacute rejection.

Regarding infectious risk, we did not observe major differences in the overall incidence of infections between the groups, although there was more fungal or parasitic infections in the no apheresis group and a clear trend toward higher CMV viremia rates in the apheresis group. No differences were observed in the occurrence of BK virus nephropathy. Theoretically, apheresis may contribute to immunosuppression by inducing hypogammaglobulinemia and hypocomplementemia, due to the non-selective nature of the technique. In a French retrospective study, 72.7% of ABO-incompatible kidney transplant recipients experienced infectious complications compared to 47.7% of ABO-compatible recipients [17]. Such differences were also reported in the literature with an increased incidence of wound infection, pneumonia, and urinary tract infection [18], and an increased rate of BK virus and CMV viremia in ABO-incompatible transplant recipients [19, 20]. Importantly, some of these infectious risks can be effectively managed with antiviral or antibiotic therapies and should not constitute a contraindication to apheresis.

Our study has several limitations. It is a retrospective analysis, and each transplant center applied its own protocol, with immunosuppressive regimens varying at the discretion of clinicians. Unfortunately, donor blood group A subtyping was not available in our centers, as this test was not part of standard clinical practice in France during the study period. Graft biopsies were interpreted according to the Banff classification in use at the time they were performed and were not centrally reviewed, introducing potential observer-related interpretation bias. Furthermore, isoagglutinin measurement techniques differed across centers. In the study by Zhou et al. [21], tube hemagglutination (used in one center in our cohort) consistently yielded lower dilution titers than gel hemagglutination, which was used in the other centers. Finally, despite the multicenter design, the sample size remains limited, and the number of events of interest was low, which may explain the lack of statistical significance in patient and graft survival outcomes when analyzed independently.

Whether outcomes in low-titer recipients differ from those observed in higher-titer ABOi patients undergoing standard desensitization remains uncertain, and the respective contributions of baseline isoagglutinin levels and pre-transplant conditioning cannot be disentangled in our study. As data on high-titer recipients were not available in our dataset, dedicated comparative studies are needed to address this question.

## Conclusion

In this multicenter French cohort of ABO-incompatible kidney transplant recipients with low baseline anti-A and/or anti-B IgG titers (≤1:8), we observed excellent overall patient and graft survival rates, highlighting the feasibility of ABO-incompatible transplantation in this selected population. However, the absence of pre-transplant apheresis was associated with lower event-free survival, and a trend toward a higher incidence of early ABMR.

Our findings suggest that even in patients with low baseline isoagglutinin titers, pre-transplant apheresis may provide additional immunological protection, potentially mitigating the risk of hyperacute or early ABMR. Nevertheless, this potential benefit must be weighed against the risk of infectious and neoplastic complications associated with intensified immunosuppression, particularly in patients also receiving depleting induction therapy. Until more sensitive and reliable assays for isoagglutinin measurement are available, it should be reasonable to consider apheresis in all ABO-incompatible recipients, regardless of baseline titer, to ensure optimal graft outcomes.

## Data Availability

The raw data supporting the conclusions of this article will be made available by the authors, without undue reservation.
