# Supplementary material for: ABO-incompatible kidney transplantation: impact of apheresis on graft and patient survival in recipients with low isoagglutinin titer
Source: Transpl Int. 2026 May 26;39:16059. doi: 10.3389/ti.2026.16059 (PMC13246458; doi:10.3389/ti.2026.16059)
Supplement: Supplementary file 2 [file Table1.docx]

| Centers | N= | Pre transplant apheresis indication | Technic | Substitution liquid | RTX | IHG target | Induction therapy* | IS protocole | Tac  Target level | Post transplant apheresis indication |
| --- | --- | --- | --- | --- | --- | --- | --- | --- | --- | --- |
| Bordeaux | 11 | No apheresis if IHG ≤ 1:8^A^ | PLEX | Albumin and AB group FFP | 375 mg/m^2^  D-30^B^ | 1:8 | ATG or basiliximab | Tac/MMF/steroids (D0-D15)  And Tac/evero/steroids (>D15) | 8-12 (D0-D15)  5-10 (>D15) | ABMR or TMA (1 patient) |
| Rouen | 9 | No apheresis if IHG<1:8, IA if IHG=1:8^C^ | Globafin | Albumin and AB group FFP | 375 mg/m^2^  D-30 | 1:8 | ATG or basiliximab | Tac/MMF/steroids | 6-8 | ABMR or TMA (2 patients) |
| Strasbourg | 9 | IA or PLEX in all cases^D^ | Therasorb or PLEX | Albumin and AB group FFP | 375 mg/m^2^  D-30 | <1:8 | ATG or basiliximab | Tac/MMF/steroids | 8-10 | ABMR or TMA (0 patient) |
| Necker | 28 | No apheresis if IHG<1:8  1 PLEX if IHG=1:8^E^ | PLEX | Albumin and AB group FFP | 375 mg/m^2^  D-30 | 1:8 | ATG or basiliximab | Tac/MMF/steroids | 7-10 | IHG ≥ 1:16 in the first 2 weeks (9 patients) |
| Nantes | 21 | No apheresis if IHG ≤ 1:16^F^ | PLEX | Albumin and AB group FFP | 200 mg/m^2^  D-15^G^ | 1:16 | ATG or basiliximab | Tac/MMF/steroids | 8-10 | ABMR or TMA (1 patient) |

Supplemental Table 1. Desensitization protocols, post-transplant immunosuppression, and indications for post-transplant apheresis across participating centers

*ATG in immunological high-risk patients, basiliximab in immunological low risk patients

^A^ 2 patients with IHG titer at 1:4 and 1:8 received PLEX sessions

^B^ 4 patients with very low IHG titer (1:1, 1:4, 1:4, 1:4) did not receive Rituximab

^C^ 3 patients received IA sessions, one for preformed DSA and 2 for IHG epuration (1/4 and 1/8 titers)

^D^ 5 patients received IA and 4 received PLEX

^E^ 4 patients without apheresis (IHG titer 0, 1:4, 1:4, 1:4), 1 patient treated by IA sessions for preformed DSA

^F^ 3 patients received PLEX sessions before transplantation (0, 1:4, 1:4 IHG titers)

^G^ One patient did not receive Rituximab for desimmunisation

IHG= immunohemaglutinin, ABMR=antibody mediated rejection, TMA= thrombotic microangiopathy, PLEX= plasma exchange, IA= immunoadsorption, FFP= fresh frozen plasma, ATG= thymoglobulin, Tac= tacrolimus, MMF= mycophenolate mofetil
