# Supplementary material for: ABO-incompatible kidney transplantation: impact of apheresis on graft and patient survival in recipients with low isoagglutinin titer
Source: Transpl Int. 2026 May 26;39:16059. doi: 10.3389/ti.2026.16059 (PMC13246458; doi:10.3389/ti.2026.16059)
Supplement: Supplementary file 7 [file Table2.docx]

| Patients (sex, age) | D/R blood group | Pre KT IHG | Nb of pre KT apheresis sessions | Preformed DSA | IHG titer at KT | Induction | DGF | TMA | ABMR | ABMR delay (d) | Post KT IHG rebound | De novo DSA | Post KT apheresis | Graft loss | Graft loss delay (y or d) |
| --- | --- | --- | --- | --- | --- | --- | --- | --- | --- | --- | --- | --- | --- | --- | --- |
| M, 39 | A🡪 B | 4 | 0 | no | 4 | ATG | Yes | Yes | Yes | 6 | No | No | Yes | No | - |
| F, 31 | O🡪 A | 4 | 0 | no | 4 | Bas. | Yes | Yes | Yes | 0 | No | No | Yes | Yes | 4.5 y |
| M , 69 | O🡪 A | 2 | 3 | Yes^*^ | 1 | Bas. | No | No | Yes | 14 | No | No | Yes | Yes | 146 d |
| M, 50 | O🡪 B | 4 | 2 | no | 8 | ATG | Yes | Yes | Yes | 0 | No | No | Yes | No | - |
| F, 49 | A🡪 B | 4 | 0 | no | 4 | ATG | Yes | Yes | Yes | 0 | No | No | Yes | No | - |
| M, 47 | O🡪 B | 0 | 0 | no | 0 | Bas. | PNF^£^ | Yes | Yes | 0 | Yes^$^ | No | Yes | Yes | 0 d |
| M, 54 | B🡪 A | 4 | 0 | no | 4 | Bas. | Yes | No | Yes | 5 | No | No | Yes | Yes | 187 d |

Supplemental Table 2. Clinical and biological description of the 7 patients who developed early acute antibody-mediated rejection and/or thrombotic microangiopathy.

^*^sum of DSA MFI= 6460

^£^ Primary non function due to cortical necrosis

^$^ IHG 1/16^e^ in the first post transplant month

D= donor, R= recipient, KT= kidney transplantation, DSA= donor specific antibody, IHG= isohemaglutinin, DGF= delayed graft function, TMA= thrombotic microangiopathy, ABMR= antibody mediated rejection, d=days, y= years, ATG= thymoglobulin, Bas.= basiliximab
