# Supplementary material for: ABO-incompatible kidney transplantation: impact of apheresis on graft and patient survival in recipients with low isoagglutinin titer
Source: Transpl Int. 2026 May 26;39:16059. doi: 10.3389/ti.2026.16059 (PMC13246458; doi:10.3389/ti.2026.16059)
Supplement: Supplementary file 8 [file Table5.docx]

Supplemental Table 5. Univariate analysis of variables associated with **biopsy-proven acute rejection** (ABMR+TCMR) in the whole cohort of transplant recipients with low IHG (n=78)

| Variable | | Coef | CI95 | p |
| --- | --- | --- | --- | --- |
| Recipient age | | 1.01 | [0.97; 1] | 0.59 |
| Recipient sex (F vs. M) |  | 1 | [0.36; 3] | 0.95 |
| Preemptive transplantation (yes vs. no) | | 0.57 | [0.2; 1.6] | 0.3 |
| First graft (yes vs. no) | | 1.5 | [0.34; 6.6] | 0.59 |
| A to O vs other (yes vs. no) | | 1.07 | [0.39; 2.9] | 0.9 |
| Donor age | | 0.99 | [0.96; 1] | 0.8 |
| Donor sex (F vs. M) |  | 0.64 | [0.23; 1.8] | 0.39 |
| Donor GFR | | 1.02 | [0.98; 1.1] | 0.32 |
| IgG at day of transplantation | |  |  |  |
|  | < 1:8 | ref. | - | - |
|  | ≥1:8 | 0.61 | [0.14; 2.7] | 0.52 |
| Induction (ATG vs. IL2RA) |  | 0.96 | [0.36; 2.6] | 0.94 |
| Rituximab (yes vs. no) | | 0.46 | [0.1; 2] | 0.3 |
| **Pre transplant Apheresis (yes vs. no)** | | **0.41** | **[0.14; 1.2]** | **0.095** |
| Sensitized patient (yes vs. no) | | 1.82 | [0.63; 5.2] | 0.27 |
| Preformed DSA (yes vs. no) | | 2.27 | [0.51; 10] | 0.28 |

GFR= glomerular filtration rate, ATG=thymoglobulin, IL2RA= anti interleukin2 receptor, IVIg= intravenous immunoglobulins, DGF= delayed graft function, DSA= donor specific antibodies
