# Supplementary material for: ABO-incompatible kidney transplantation: impact of apheresis on graft and patient survival in recipients with low isoagglutinin titer
Source: Transpl Int. 2026 May 26;39:16059. doi: 10.3389/ti.2026.16059 (PMC13246458; doi:10.3389/ti.2026.16059)
Supplement: Supplementary file 9 [file Table3.docx]

| Patients | Apheresis Group | Cause of graft loss | Rejection related | Delay of graft loss (years-months) |
| --- | --- | --- | --- | --- |
| 41 | No apheresis | Repeated sepsis, ATN | No | 3y 5m |
| 58 | Apheresis | Mixed rejection, no DSA | Yes | 2y 10m |
| 73 | No apheresis | Chronic rejection, no DSA | Yes | 4y 6m |
| 77 | Apheresis | IgAN recurrence | No | 4y 4m |
| 78 | Apheresis | Preformed DSA, ABMR, collapsing FSGS | Yes | 5m |
| 93 | No apheresis | IgAN recurrence | No | 2y 4m |
| 117 | No apheresis | Mixed rejection, dn DSA | Yes | 2y 11m |
| 126 | No apheresis | Mixed rejection, dn DSA | Yes | 3y |
| 153 | No apheresis | Cortical necrosis, TMA | Yes | 0 |
| 184 | No apheresis | Hematoma, urinoma and ABMR | Yes | 4m |

Supplemental Table 3. Causes of graft loss in the 10 patients of the cohort

ATN= acute tubular necrosis, NTX= nephrotoxicity, DSA= donor specific antibody, dn DSA= de novo donor specific antibody, IgAN= IgA nephropathy, FSGS= focal segmental glomerulosclerosis, ABMR= antibody mediated rejection, TMA= thrombotic microangiopathy, m=months, y= years
