# Supplementary material for: ABO-incompatible kidney transplantation: impact of apheresis on graft and patient survival in recipients with low isoagglutinin titer
Source: Transpl Int. 2026 May 26;39:16059. doi: 10.3389/ti.2026.16059 (PMC13246458; doi:10.3389/ti.2026.16059)
Supplement: Supplementary file 10 [file Table4.docx]

| Variable | | | | Coef | CI95 | p |
| --- | --- | --- | --- | --- | --- | --- |
| Recipient age | | | | 1 | [0.96; 1] | 1 |
| Recipient sex (F vs. M) | |  | 1.3 | | [0.43; 3.9] | 0.65 |
| Preemptive transplantation (yes vs. no) | | | | 0.5 | [0.16; 1.6] | 0.24 |
| First graft (yes vs. no) | | | | 1.27 | [0.28; 5.7] | 0.75 |
| A to O vs other | | | | 0.68 | [0.21; 2.2] | 0.52 |
| Donor age | | | | 0.99 | [0.95; 1] | 0.63 |
| Donor sex (F vs. M) |  | | | 0.5 | [0.17; 1.5] | 0.2 |
| Donor GFR | | | | 1.03 | [0.99; 1.1] | 0.097 |
| IHG day of transplantation | | | |  |  |  |
|  | < 1:8 | | | ref. | - | - |
|  | ≥1:8 | | | 0.73 | [0.16; 3.3] | 0.68 |
| Induction (ATG vs. IL2RA)) |  | | | 1.4 | [0.47; 4.1] | 0.56 |
| Rituximab (yes vs. no) | | | | 0.92 | [0.12; 7] | 0.93 |
| **Pre transplant apheresis (yes vs. no)** | | | | **0.36** | **[0.11; 1.1]** | **0.081** |
| Sensitized patient (yes vs. no) | | | | 2.04 | [0.64; 6.5] | 0.23 |
| Preformed DSA (yes vs. no) | | | | 2.67 | [0.59; 12] | 0.2 |

Supplemental Table 4. Univariate analysis of variables associated with **antibody mediated rejection** in the whole cohort of transplant recipients with low IHG (n=78)

IHG= isohemagglutinin, GFR= glomerular filtration rate, ATG=thymoglobulin, IL2RA= anti interleukin2 receptor, IVIg= intravenous immunoglobulins, DGF= delayed graft function, DSA= donor specific antibodies
